# Validation of an Aptima-format Finnish new variant of *Chlamydia trachomatis* (FI-nvCT) surveillance assay, 2019

**DOI:** 10.2807/1560-7917.ES.2020.25.5.2000038

**Published:** 2020-02-06

**Authors:** Barbara Weinbaum, Analee Williams, Ronza Hadad, Bryan Vinluan, Mirja Puolakkainen, Magnus Unemo, Damon Getman

**Affiliations:** 1 Research and Development, Hologic Inc., San Diego, United States; 2 World Health Organization Collaborating Centre for Gonorrhoea and Other Sexually Transmitted Infections (STIs), National Reference Laboratory for STIs, Department of Laboratory Medicine, Faculty of Medicine and Health, Örebro University, Örebro, Sweden; 3 Department of Virology and Immunology, University of Helsinki and Helsinki University Hospital, HUSLAB, Helsinki, Finland

**Keywords:** Finish new variant, Chlamydia trachomatis, Aptima Combo 2, surveillance

## Abstract

The Finnish new variant of *Chlamydia trachomatis* (FI-nvCT) is escaping diagnostics in Finland, Norway and Sweden. We have developed and validated an Aptima-format nucleic acid amplification test (NAAT) designed specifically to detect the FI-nvCT. This NAAT has high sensitivity (100%) and specificity (100%) for the FI-nvCT strain, enabling further investigation of the geographic distribution, prevalence and transmission of this diagnostic-escape mutant in screening populations in Europe.

In February 2019, investigation of discrepant nucleic acid amplification test (NAAT) results led to the discovery of a new genetic variant strain of *Chlamydia trachomatis* in south-western Finland [1]. This strain, designated Finnish new variant of *C. trachomatis* (FI-nvCT), harbours a single nt base mutation (C1515T; *Escherichia coli* numbering) in 23S rRNA. This mutation was determined to be the root cause for compromised detection of the organism by the Aptima Combo 2 (AC2) diagnostic test (Hologic Inc., San Diego, California, United States (US)), which targets *C. trachomatis* 23S rRNA [2,3]. Archived and newly received clinical specimens in Finland and Sweden with equivocal results or high-negative relative light units (RLU) from 15 to 99 in the AC2 test, and discrepant results between AC2 (target: 23S rRNA) and the separate Aptima *Chlamydia trachomatis* (ACT) test (target: 16S rRNA) (i.e. AC2 negative/equivocal—ACT positive) signalled the presence of the new strain. Data collected in subsequent investigations suggested a recent emergence of FI-nvCT in south-western Finland, Norway and Sweden with relatively low prevalence (ca 1–6%) among patients with *C. trachomatis* infections [1,3-5]. We have developed and validated a research-use only Aptima-format surveillance NAAT designed specifically to detect the FI-nvCT strain. This assay should facilitate further investigations to determine the geographic distribution, prevalence and transmission of this diagnostic-escape mutant.

## New Aptima-format surveillance assay for the Finnish new variant of *Chlamydia trachomatis*

To understand the geographic distribution, prevalence and transmission dynamics of the FI-nvCT strain nationally and internationally, we have developed an Aptima-format NAAT that sensitively and specifically detects FI-nvCT (23S rRNA gene C1515T mutation) but not wild-type (C1515) *C. trachomatis* strains. This research-use only test uses target capture and transcription-mediated amplification chemistries on the automated Panther instrument (Hologic Inc.) to isolate and amplify *C. trachomatis* 23S rRNA, and an acridinium ester probe to selectively detect the C→T mutation at position 1515.

## Validation and verification results for the Aptima-format surveillance assay for the Finnish new variant of *C. trachomatis*

**Figure 1:**
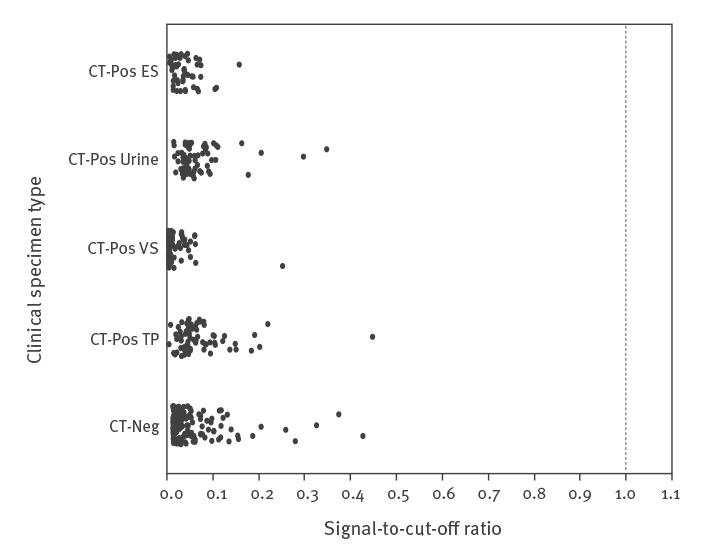
Clinical specificity of the Aptima-format Finnish new variant of *Chlamydia trachomatis* (FI-nvCT) surveillance assay on the Panther instrument^a^, United States 2019

CT: *Chlamydia trachomatis*; ES: endocervical swab; Neg: negative; Pos: positive; TP: PreservCyt ThinPrep liquid cytology; VS: vaginal swab.

^a^ Signal-to-cut-off ratio values for the FI-nvCT assay are from testing clinical specimens obtained from a United States reference laboratory that are positive (n = 266) or negative (n = 188) for *C. trachomatis* by the Aptima Combo 2 assay. Of the 266 positive specimens: ES (n = 54); male and female urine (n = 71); VS (n = 68); TP (n = 73).

All validation and verification studies of the new FI-nvCT assay were conducted according to Clinical Laboratory Standards Institute (CLSI) standard protocols used for in vitro diagnostic assay validation [6]. The cut-off value of the test (100,000 RLU) was initially established by testing 454 de-identified remnant clinical specimens obtained from a large reference laboratory in the US. The collection consisted of 266 specimens (endocervical and vaginal swabs, male and female urine, PreservCyt ThinPrep liquid cytology) positive for *C. trachomatis* by the AC2 assay, and 188 specimens (mixed types) that were AC2-negative (Figure 1). Signal-to-cut-off (S/CO) ratio values for all clinical specimens tested ranged from 0 to 0.45. Mean S/CO ratio values ranged from 0.02 to 0.071 for *C. trachomatis* C1515-positive (wild type) specimens, and equalled 0.046 for specimens negative for *C. trachomatis.* Negative controls included in this analysis (n = 6), consisting of 10,000 copies/mL of wild type (C1515) *C. trachomatis* RNA, yielded S/CO ratio values ranging from 0.016 to 0.112 (mean: 0.044); positive controls (n = 6) consisting of 1,000 copies/mL of IVT RNA corresponding to FI-nvCT (C1515T) rRNA yielded S/CO ratio values ranging from 3.60 to 9.02 (mean: 6.38) (data not shown).

The dotted line represents the cut-off value established for the FI-nvCT surveillance assay.

**Figure 2:**
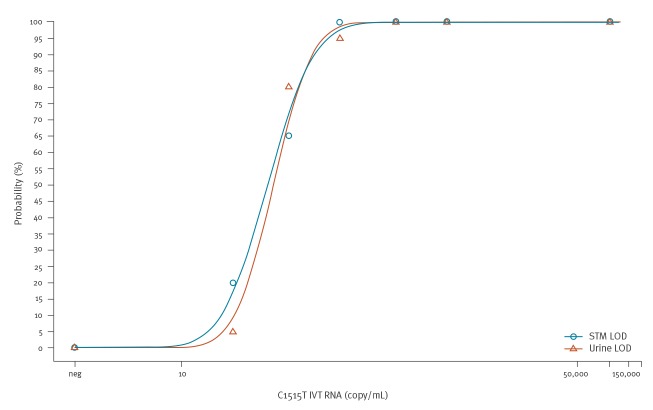
Analytical sensitivity as Probit 95% LOD of the Aptima-format Finnish new variant of *Chlamydia trachomatis* (FI-nvCT) surveillance assay on the Panther instrument, United States, 2019

IVT: in vitro transcript; LOD: limit of detection; neg: negative; STM: sample transport medium.

The analytical sensitivity of the new Aptima-format FI-nvCT surveillance assay for detecting in vitro transcript (IVT) RNA containing the *C. trachomatis* 23S rRNA C1515T mutation using the 100,000 RLU cut-off was established by Probit analysis of a panel of six positive and one negative FI-nvCT RNA samples (n = 20 replicates each panel member) (Figure 2). This analysis established the 95% limit of detection (LOD) for the assay is 231 copies/mL in Aptima sample transport medium and 210 copies/mL in urine sample matrix, equivalent to ca 0.072 infectious units per mL (IFU/mL) and 0.065 IFU/mL, respectively (copies to IFU conversion data not shown).

Probit 95% LOD values for detection of RNA containing the C1515T sequence are 210 copies/mL for urine specimens, and 231 copies/mL for sample transport medium (STM) specimens.

Analytical specificity (cross-reactivity) and interference (spike and recovery) of the assay against non-target organisms potentially encountered in urogenital or extra-genital specimens was assessed by testing a minimum of ten replicates of each of 22 pools consisting of 3 to 4 organisms per pool, encompassing 59 bacterial, 2 yeast, 4 protozoal and 19 viral species at high titre (Table 1). All non-target organisms tested yielded negative results in the assay. To assess non-target organism interference, 23S rRNA C1515T IVT RNA was spiked to a concentration of three times the assay 95% LOD (660 copies/mL) and tested in the presence of the same high titre pools. All results were positive for the 23S rRNA C1515T-spiked specimens (Table 1) except for Panel 20, which was 95% positive. In addition, culture isolates of *C. trachomatis* serotypes A, B, Ba, C, D, E, F, G, H, I, J, K, L1, L2 and L3 (all containing the wild type base C1515 in the 23S rRNA) were tested at 1x10^5^ IFU/mL each (except serotype G, which was tested at 3x10^4^ IFU/mL). All serotypes tested (n = 5 replicates each) yielded negative results in the assay with S/CO ratio values ranging from 0.129 to 0.248 (data not shown).

**Table 1:** Cross reactivity and interference testing of non-target organisms with the Aptima-format Finnish new variant of *Chlamydia trachomatis* (FI-nvCT) surveillance assay on the Panther instrument, United States, 2019

Pool	Organism	Concentration	FI-nvCT negative		FI-nvCT RNA positive (3X LOD)	
			Number Positive	S/CO	Number Positive	S/CO
1	*Acinetobacter lwoffii*	1e6 CFU/mL	0/10	0.048	10/10	2.015
	*Actinomyces israelii*	1e6 CFU/mL				
	*Alcaligenes faecalis*	1e6 CFU/mL				
	*Anaerococcus vaginalis*	1e6 CFU/mL				
2	*Arcanobacterium haemolyticum*	1e6 CFU/mL	0/10	0.036	10/10	2.635
	*Atopobium vaginae*	1e6 CFU/mL				
	*Bacteroides fragilis*	1e6 CFU/mL				
	*Bacteroides oralis*	1e6 CFU/mL				
3	*Bifidobacterium adolescentis*	1e6 CFU/mL	0/10	0.026	10/10	5.769
	*Bordatella parapertussis*	1e6 CFU/mL				
	*Campylobacter jejuni*	1e6 CFU/mL				
	*Campylobacter rectus*	1e6 CFU/mL				
4	*Citrobacter koseri*	1e6 CFU/mL	0/10	0.035	10/10	4.679
	*Corynebacterium diphtheria*	1e6 CFU/mL				
	*Corynebacterium genitalium*	1e6 CFU/mL				
	*Corynebacterium pseudodiptheriticum*	1e6 CFU/mL				
5	*Eggerthella lenta*	1e6 CFU/mL	0/10	0.050	10/10	5.638
	*Enterobacter cloacae*	1e6 CFU/mL				
	*Enterococcus faecalis*	1e6 CFU/mL				
	*Escherichia coli*	1e6 CFU/mL				
6	*Fusobacterium necrophorum*	1e6 CFU/mL	0/10	0.043	10/10	5.705
	*Fusobacterium nucleatum*	1e6 CFU/mL				
	*Gardnerella vaginalis*	1e6 CFU/mL				
	*Helicobacter pylori*	1e6 CFU/mL				
7	*Haemophilus ducreyi*	1e6 CFU/mL	0/10	0.036	10/10	5.485
	*Haemophilus parahaemolyticus*	1e6 CFU/mL				
	*Haemophilus parainfluenzae*	1e6 CFU/mL				
	*Klebsiella pneumoniae*	1e6 CFU/mL				
8	*Lactobacillus acidophilus*	1e6 CFU/mL	0/10	0.042	10/10	4.628
	*Legionella (Tatlockia) micdadei*	1e6 CFU/mL				
	*Legionella jordanis*	1e6 CFU/mL				
	*Leptotrichia buccalis*	1e6 CFU/mL				
9	*Listeria monocytogenes*	1e6 CFU/mL	0/10	0.061	10/10	4.554
	*Megasphaera* type 1	1e6 CFU/mL				
	*Mobiluncus curtisii*	1e6 CFU/mL				
	*Moraxella catarrhalis*	1e6 CFU/mL				
10	*Mycoplasma genitalium*	1e6 CFU/mL	0/10	0.034	10/10	5.514
	*Mycoplasma hominis*	1e6 CFU/mL				
	*Mycoplasma pneumoniae*	1e4 CFU/mL				
	*Neisseria gonorrhoeae*	1e6 CFU/mL				
11	*Peptostreptococcus micros*	1e6 CFU/mL	0/10	0.041	10/10	3.074
	*Propionibacterium acnes*	1e6 CFU/mL				
	*Staphylococcus aureus*	1e6 CFU/mL				
	*Staphylococcus epidermidis*	1e6 CFU/mL				
12	*Proteus vulgaris*	1e6 CFU/mL	0/10	0.031	10/10	5.617
	*Shigella dysenteriae*	1e6 CFU/mL				
	*Shigella flexneri*	1e6 CFU/mL				
	*Shigella sonneri*	1e6 CFU/mL				
13	*Stenotrophomonas maltophilia*	1e6 CFU/mL	0/10	0.020	10/10	6.025
	*Streptococcus agalactiae*	1e6 CFU/mL				
	*Streptococcus anginosus*	1e6 CFU/mL				
	*Streptococcus pyogenes*	1e6 CFU/mL				
14	*Ureaplasma parvum*	1e6 CFU/mL	0/10	0.038	10/10	3.971
	*Ureaplasma urealyticum*	1e6 CFU/mL				
	*Veillonella parvula*	1e6 CFU/mL				
	*Burkholderia cepacia*	1e6 CFU/mL				
15	*Clostridium difficile*	1e6 CFU/mL	0/10	0.040	10/10	3.991
	*Prevotella bivia*	1e6 CFU/mL				
	*Candida albicans*	1e6 CFU/mL				
	*Cryptococcus neoformans*	1e6 CFU/mL				
16	*Entamoeba histolytica*	1e4 cells/mL	0/10	0.052	10/10	3.963
	*Giardia lamblia*	1e4 cells/mL				
	*Pentatrichomonas hominis*	1e5 units/mL				
	*Trichomonas vaginalis*	1e5 cells/mL				
17	Adenovirus type 07A	1e5 TCID50/mL	0/10	0.029	10/10	5.076
	Coronavirus 229E	1e5 TCID50/mL				
	Coxsackievirus B3	1e5 TCID50/mL				
	Echovirus type 11	1e5 TCID50/mL				
18	Enterovirus type 68	1e4 TCID50/mL	0/10	0.060	19/20	4.349
	Epstein-Barr virus	1e6 copies/mL				
	Hepatitis B virus	1e6 IU/mL				
	Hepatitis C virus	1e5 IU/mL				
19	HIV	1e5 copies/mL	0/10	0.073	37/40^a^	1.902
	HPV 16 (SiHa cells)	1e4 cells/mL				
	HPV 18 (HeLa cells)	1e4 cells/mL				
	Human metapneumovirus type 20	1e6 TCID50/mL				
20	HSV I	1e4 TCID50/mL	0/10	0.047	19/20	4.225
	HSV II	1e4 TCID50/mL				
	Influenza A(H3N2)	1e3 TCID50/mL				
	Influenza B Massachusetts/2/12	1e3 TCID50/mL				
21	Norovirus Group II	1e6 TCID50/mL	0/10	0.082	10/10	3.592
	Respiratory syncytial virus type B	1e5 TCID50/mL				
	Rhinovirus A16	1e5 TCID50/mL				
22	*Chlamydia pneumoniae*	1e5 IFU/mL	0/10	0.033	10/10	3.219
	*Chlamydia psittaci* (GP1802)	1e5 IFU/mL				
	*Chlamydia psittaci* (GP1557)	1e5 IFU/mL				
23	Control pool negative	NA	0/15	0.031	NA	NA
24	Control pool positive	NA	NA	NA	15/15	7.14

CFU: colony forming unit; HMPV: human metapneumovirus; HPV: human papillomavirus; HSV: herpes simplex virus; IFU: inclusion forming unit; LOD: limit of detection: NA: not applicable; S/CO; signal-to-cut-off; TCID50: tissue culture infectious dose 50.

^a^ Initial result at 37/40 (93%). Organisms in the panel were tested individually with FI-nvCT (n = 10 each) and were 10/10 (100%) positive for each.

S/CO: HIV: 6.171; HPV16: 2.537; HPV18: 6.396; HMPV: 4.894.

## European validation of the Aptima-format surveillance assay for the Finnish new variant of *C. trachomatis*

Additional validation studies of the Aptima-format FI-nvCT surveillance assay using spiked specimens and clinical specimens was performed at the World Health Organization (WHO) Collaborating Centre for Gonorrhoea and other Sexually Transmitted Infections in Örebro, Sweden. The results of this validation are summarised in Table 2. Briefly, all 10 WHO *Neisseria gonorrhoeae* reference strains; 153 isolates of 14 non-*N. gonorrhoeae* *Neisseria* species and three *Moraxella* species; 10 *Trichomonas vaginalis* (TV) samples, including the two TV ATCC strains 30001 and 50140; five reference strains of four non-TV *Trichomonas* species; nine *C. trachomatis* strains of different genotypes and the Swedish new variant of *C. trachomatis* (SE-nvCT); four non-*C. trachomatis* *Chlamydia* species; 428 clinical *C. trachomatis* wild type positive AC2 specimens; 23 clinical specimens positive for *N. gonorrhoeae*, *T. vaginalis*, bacterial vaginosis and *Candida* species; and 443 clinical AC2-negative specimens, were negative in the FI-nvCT assay. However, all 69 FI-nvCT positive clinical AC2 specimens (verified by 23S rRNA gene sequencing) were positive in the new Aptima-format FI-nvCT surveillance assay (Table 2).

**Table 2:** Cross reactivity of non-target organisms (spiked samples) and detection in clinical specimens of the Aptima-format Finnish new variant of *Chlamydia trachomatis* (FI-nvCT) surveillance assay on the Panther instrument, Europe, 2019

Bacterial species or specimen	Number tested	FI-nvCT positive	FI-nvCT negative
*Neisseria gonorrhoeae* WHO F, G, L, M, U, V, W, X, Y and Z	10	0	10
*N. gonorrhoeae*-positive AC2 specimens	5	0	5
*N. animalis*	1	0	1
*N. bergeri*	1	0	1
*N. cinerea*	9	0	9
*N. elongata*	3	0	3
*N. flava*	1	0	1
*N. flavescens*	90	0	90
*N. lactamica*	12	0	12
*N. macacae*	17	0	17
*N. mucosa*	18	0	18
*N. oralis*	1	0	1
*N. perflava*	62	0	62
*N. sicca*	9	0	9
*N. subflava*	6	0	6
*N. meningitidis* ^a^	17	0	17
*Moraxella catarrhalis*	1	0	1
*M. nonliquefaciens*	1	0	1
*M. osloensis*	1	0	1
*Trichomonas vaginalis* (ATCC 30001 and ATCC 50140)	10	0	10
*T. vaginalis*-positive AC2 specimens	8	0	8
*T. aotus* (ATCC 50649)	1	0	1
*T. gallinae* (ATCC 30002, 30230)	2	0	2
*T. stableri* (ATCC PRA-412)	1	0	1
*T. tenax* (ATCC 30207)	1	0	1
Bacterial vaginosis-positive vaginal swabs	5	0	5
*Candida* spp.-positive vaginal swabs	5	0	5
*C. trachomatis* Ba, D, E, F, G, H, J, K and L2b	9	0	9
*C. trachomatis* wild type (C1515)-positive clinical AC2 specimens	428	0	428
*C. trachomatis* SE-nvCT	1	0	1
*C. suis* (ATCC VR-1474)	1	0	1
*C. muridarum* (ATCC VR-123)	1	0	1
*Chlamydia pneumoniae* (ATCC VR-2282, MBC011)	2	0	2
*Chl. psittaci* (MBC013)	1	0	1
*C. trachomatis* and *N. gonorrhoeae*-negative clinical AC2 specimens	443	0	443
FI-nvCT-positive clinical AC2 specimens^b^	69	69	-

ATCC: American Type Culture Collection; SE-nvCT: Swedish new variant of *Chlamydia trachomatis*; AC2: Aptima Combo 2; WHO: World Health Organization.

^a^ Representing all major meningococcal clones spreading worldwide, including serogroups A, B, C, E, W, X, Y and Z.

^b^ Confirmed by sequencing of the 23S rRNA gene.

## Discussion

This study presents validation and verification data for a new research-use Aptima-format NAAT for detection of the FI-nvCT strain. The assay selectively detects with high sensitivity (100%) and specificity (100%) *C. trachomatis* strains containing 23S rRNA with the C1515T mutation found to be the root cause of compromised detection in the AC2 assay and does not detect *C. trachomatis* strains lacking this nt variance.

Genetic variation in *C. trachomatis*, a highly prevalent bacterial STI [7], has historically been considered uncommon. However, a recent large-scale genomic analysis of geographically and temporally diverse *C. trachomatis* strains revealed a remarkable degree of homologous recombination and mutations that have occurred in the organism in the recent millennia [8]. This new understanding provides additional insight into the previous discovery in 2006 of a diagnostic-escape mutant of *C. trachomatis* that spread undetected in Sweden for 3 to 4 years before its discovery [9-12]. This SE-nvCT has a deletion in the plasmid DNA targeted by two widely used diagnostic NAATs in use at that time [9], which allowed the SE-nvCT strain to reach high prevalence levels (> 60%) in some segments of the infected population [10].

The automated research-use only Aptima-format FI-nvCT surveillance test described here should facilitate the rapid completion of epidemiologic surveillance studies to estimate FI-nvCT prevalence and to track the geographic and temporal spread of this new diagnostic-escape variant in people screened for STIs in Europe.
